# Supplementary material for: Subunit interactions and arrangements in the fission yeast Mis16–Mis18–Mis19 complex
Source: Life Sci Alliance. 2019 Aug 1;2(4):e201900408. doi: 10.26508/lsa.201900408 (PMC6677171; doi:10.26508/lsa.201900408)
Supplement: Supplementary file 1 [file LSA-2019-00408_TableS1.docx]

|  | **Mis16** | **Mis16-H4** | **Mis16-Mis19** |
| --- | --- | --- | --- |
| Wavelength (Å) | 0.97795 | 0.8726 | 1.00004 |
| Resolution range (Å) | 51.68 - 1.94 (2.009 - 1.94) | 46.22 - 1.8 (1.864 - 1.8) | 54.28 - 1.988 (2.06 - 1.988) |
| Space group | C 2 2 2(1) | P 2(1) 2(1) 2(1) | P 2(1) |
| Unit cell (Å, degrees) | 75.7968 123.604 94.2438 90 90 90 | 57.39 77.98 97.51 90 90 90 | 53.1711 99.035 109.88 90 98.8663 90 |
| Total reflections | 66260 (6572) | 79190 (7629) | 389953 (7431) |
| Unique reflections | 33131 (3286) | 39885 (3857) | 69320 (3761) |
| Multiplicity | 2.0 (2.0) | 2.0 (2.0) | 5.6 (2.0) |
| Completeness (%) | 99.92 (99.85) | 96.58 (94.95) | 89.56 (48.69) |
| Mean I/sigma(I) | 8.38 (0.83) | 9.36 (0.89) | 8.11 (1.26) |
| Wilson B-factor | 36.75 | 29.89 | 26.27 |
| R-merge | 0.03302 (0.7461) | 0.04434 (0.8191) | 0.1481 (0.623) |
| R-meas | 0.0467 (1.055) | 0.0627 (1.158) | 0.1623 (0.8069) |
| R-pim | 0.03302 (0.7461) | 0.04434 (0.8191) | 0.06497 (0.5044) |
| CC1/2 | 0.999 (0.496) | 0.998 (0.316) | 0.993 (0.611) |
| CC* | 1 (0.814) | 1 (0.693) | 0.998 (0.871) |
| Reflections used in refinement | 33107 (3281) | 39872 (3857) | 69226 (3751) |
| Reflections used for R-free | 1630 (163) | 1921 (174) | 3556 (193) |
| R-work | 0.2041 (0.2989) | 0.2041 (0.3615) | 0.1959 (0.2660) |
| R-free | 0.2597 (0.3270) | 0.2406 (0.3978) | 0.2341 (0.3167) |
| CC(work) | 0.957 (0.692) | 0.962 (0.554) | 0.943 (0.810) |
| CC(free) | 0.918 (0.673) | 0.940 (0.524) | 0.927 (0.687) |
| Number of non-hydrogen atoms | 3155 | 3480 | 7945 |
| macromolecules | 3029 | 3224 | 7163 |
| ligands |  |  | 6 |
| solvent | 126 | 256 | 776 |
| Protein residues | 380 | 406 | 894 |
| RMS(bonds, Å) | 0.008 | 0.008 | 0.009 |
| RMS(angles, degrees) | 1.29 | 1.25 | 1.11 |
| Ramachandran favored (%) | 96.79 | 96.73 | 97.15 |
| Ramachandran allowed (%) | 2.94 | 2.76 | 2.28 |
| Ramachandran outliers (%) | 0.27 | 0.5 | 0.57 |
| Rotamer outliers (%) | 0 | 0 | 0.13 |
| Clashscore | 5.74 | 5.06 | 3.90 |
| Average B-factor | 43.41 | 35.81 | 31.66 |
| macromolecules | 43.32 | 35.4 | 31.18 |
| ligands |  |  | 30.61 |
| solvent | 45.64 | 40.87 | 36.10 |

Supplementary Table 1
